# CD36^+^ Fibroblasts Secrete Protein Ligands That Growth-Suppress Triple-Negative Breast Cancer Cells While Elevating Adipogenic Markers for a Model of Cancer-Associated Fibroblast

**DOI:** 10.3390/ijms232112744

**Published:** 2022-10-22

**Authors:** Kosar Jabbari, Qingsu Cheng, Garrett Winkelmaier, Saori Furuta, Bahram Parvin

**Affiliations:** 1 Department of Biomedical Engineering, University of Nevada, Reno, NV 89557, USA; 2 Department of Biomedical Engineering, University of Wisconsin-Milwaukee, Milwaukee, WI 53211, USA; 3 Department of Cancer Biology, University of Toledo Health Sciences Campus, Toledo, OH 43614, USA; 4 Pennington Cancer Institute, Renown Health, Reno, NV 89502, USA

**Keywords:** CD36^+^ fibroblasts, tumor suppression, cancer-associated fibroblasts, stromal reprogramming

## Abstract

Tumor and stroma coevolve to facilitate tumor growth. Hence, effective tumor therapeutics would not only induce growth suppression of tumor cells but also revert pro-tumor stroma into anti-tumoral type. Previously, we showed that coculturing triple-negative or luminal A breast cancer cells with CD36^+^ fibroblasts (FBs) in a three-dimensional extracellular matrix induced their growth suppression or phenotypic reversion, respectively. Then, we identified SLIT3, FBLN-1, and PENK as active protein ligands secreted from CD36^+^ FBs that induced growth suppression of MDA-MB-231 breast cancer cells and determined their minimum effective concentrations. Here, we have expanded our analyses to include additional triple-negative cancer cell lines, BT549 and Hs578T, as well as HCC1937 carrying a BRCA1 mutation. We show that the ectopic addition of each of the three ligands to cancer-associated fibroblasts (CAFs) elevates the expression of CD36, as well as the adipogenic marker FABP4. Lastly, we show that an agonist antibody for one of the PENK receptors induces growth suppression of all cancer cell lines tested but not for non-transformed MCF10A cells. These results clearly suggest that proteins secreted from CD36^+^ FBs induce not only growth suppression of tumor cells through binding the cognate receptors but also increasing adipogenic markers of CAFs to reprogram tumor stroma.

## 1. Introduction

Stromal and parenchymal components of a tissue, whether healthy or malignant, coevolve and depend on each other for their survival [1,2,3]. One of the stromal components that have a major impact on cancer progression is fibroblasts (FBs). It has been suggested that “the next 10 years warrants to be an exciting time for unraveling more hidden secrets of FBs” [4]. For example, standard chemotherapies often promote the emergence of cancer-associated fibroblasts (CAFs) [5,6]. Although CAF biomarkers lack standardization [7], CAFs largely contribute to the pro-tumor microenvironment [5] and multidrug resistance (MDR), which accounts for approximately 90% of cancer-related mortality [8]. It is anticipated that the next generation of cancer therapeutics will likely leverage the body’s own intrinsic and natural responses. Hence, we have sought to trigger tumor suppression by restoring the normal epithelia–FB communications. Here, we have extended our previous findings that FBs expressing CD36, a receptor initially discovered through its roles in lipid uptake and adipogenesis, secrete a cocktail of protein ligands that inhibit the growth of certain types of breast cancer [9,10]. CD36 is expressed in FBs of normal mammary glands [9], where normal mammary FBs are known to exert anti-tumor functions through paracrine signaling [11,12]. Downregulation of CD36 in FBs is one of the CAF markers [5], and in the normal mammary gland, it is often associated with high mammographic density (MD) [13]. Conversely, CD36 is often overexpressed in cancer (epithelial) cells and associated with worse clinical outcomes [14]. Such apparently opposing effects of CD36 expression in cancer cells vs. CAFs make it difficult to target CD36 directly for cancer therapy. We then investigated whether factors secreted from CD36^+^ FBs might exert anti-tumor effects. We showed that coculturing triple-negative or luminal A breast cancer cells with CD36^+^ FBs in 3D ECM effectively inhibited cancer cell growth [10]. Next, we performed comparative proteomic profiling of the secretome of CD36^+^ vs. CD36^−^ FBs and identified a number of candidate protein ligands. After the functional screening, we narrowed down active ligands to SLIT3, PENK, and FBLN-1, and determined their minimum effective concentrations [9]. In the present study, we show that each of the three ligands could also reprogram CAFs and induce their transdifferentiation by overexpressing CD36 and FABP4 while strongly suppressing the growth of breast cancer cells. These results suggest that these ligands could be utilized to develop a new type of breast tumor therapy that simultaneously targets both tumor cells and tumor stroma.

## 2. Results

### 2.1. CD36^+^ Conditioned Medium Has Minimal Effect on Colony Formation of Non-Transformed MCF10A Cell Line

Earlier, we showed that the coculture of the triple-negative breast cancer (TNBC) MDA-MB-231 or Luminal A MCF7 with CD36^+^ fibroblasts (FBs) induced growth suppression in TNBC MDA-MB-231 and reversion of basal and lateral polarity in the Luminal A MCF7 [10]. Subsequently, we also showed that the conditioned medium (CM) of the CD36^+^ fibroblasts (FBs) also induces growth suppression in MDA-MB-231 [9]. Because the CM can be concentrated and used as a positive control for future comparative studies, it is important to examine the impact of its concentration on colony formation for a non-transformed cell line such as MCF10A, which is a mammary epithelial cell line. This result is shown in Supplementary Figure S1, which indicates a reduction of approximately 30% in colony formation with the highest concentration of CD36^+^ CM, where the ratio of MCF10A to FB is 1:10. However, the reduction in colony formation is largely due to the culture condition. The fibroblast medium (DMEM) differs from the MCF10A medium (DMEM/F12 + supplements), and adding the CD36^+^ CM decreased the concentration of necessary supplements for MCF10A growth. Hence, as an extra control, the equivalent amount of DMEM (not conditioned by FBs) is added to the MCF10A culture to clarify the effect of the vehicle and is represented by Control+DMEM.

### 2.2. Recombinant Protein Ligands Induce Growth Suppression in Triple-Negative Breast Cancer Cell Lines

To start, neutralizing antibodies for SLIT3, FBLN1, and PENK at concentrations of 1µg/mL were added to the MDA-MB-231 growth medium with and without the CM of the CD36^+^ FBs. Each neutralizing antibody is added one at a time and then three at a time, with the results shown in Supplementary Figure S2. The results indicated that the growth suppression was reverted by the addition of each of the neutralizing antibodies and reverted more by the mixture of the three neutralizing antibodies. Although growth suppression by other factors in the CM of CD36^+^ FBs cannot be ruled out, it is evident that a significant factor of growth suppression is associated with SLIT3, FBLN1, and PENK protein ligands.

**Figure 1:**
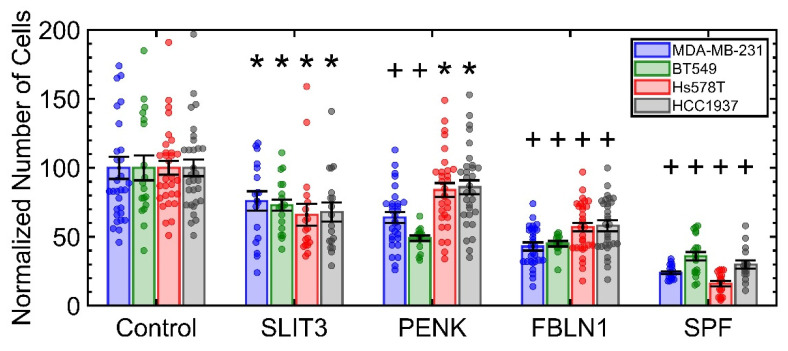
Each of the recombinant proteins induces growth suppression in all four TNBC cell lines. Their cocktail (SPF) induces higher growth suppression, which suggests an additive effect. All statistics are computed in reference to the control. Each condition had a minimum of six replicates with at least two fields of view per well imaged. * *p*-value < 0.05 and + *p*-value < 0.001.

Next, growth suppression in four TNBC lines of MDA-MB-231, Hs578T, BT549, and HCC1937 was quantified for each of the recombinant proteins (RPs) or their cocktail (represented as SPF), with the results shown in Figure 1. MDA-MB-231, Hs578T, and BT549 are classified as Basal B [15] and form a stellate phenotype in 3D culture [16]. HCC1937 has BRCA1 mutation and forms round colonies in 3D [17], with an example shown in Supplementary Figure S3. Regardless, the sensitivity of these cell lines to RPs did not appear to be cell-line specific, and in all cases, the cocktail of ligands showed higher growth suppression, which suggests that these recombinant proteins have an additive growth suppression effect. The control for this experiment was non-malignant MCF10A which showed an approximately 20% reduction in the rate of colony formation as per our previous manuscript (Supplementary Results) [9].

### 2.3. Recombinant SLIT3 Elevates the Expression of Its Receptor, ROBO2, Suggesting a Positive Feedback Loop for Tumor Suppression in TNBC Lines

**Figure 2:**
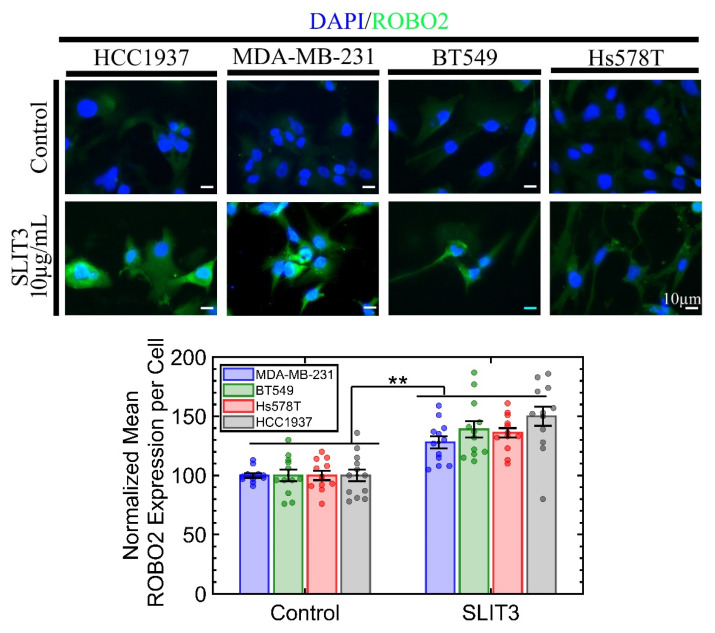
ROBO2 is overexpressed in all four TNBC cell lines when these cells are incubated with SLIT3. Hence, the ectopic SLIT3 reprograms cancer cells. Each condition had six replicates with at least two fields of view per well imaged. ** *p*-value < 0.001.

The SLIT3 protein consists of regions that include an N-terminal signaling peptide, four leucine-rich repeat domains, six EGF domains, and a laminin G domain followed by a cysteine-rich C-terminal region [18]. All SLIT3 proteins can be cleaved between the fifth and sixth EGF-like domains into the N-terminal (140 kDa) and shorter C-terminal (50–60 kDa) segments [19]. ROBO is a transmembrane (TM) protein with five immunoglobulin folds, three fibronectin type III repeats, a TM domain, and four conserved cytoplasmic motifs in its intracellular domain [20]. SLIT3 is commercially available with N-terminus and C-terminus fragments. The N-terminus, from Novus Biological, is produced in HEK293 cells, whereas the C-terminus from Abbexa (Cambridge, UK) is produced from *E. coli*. Both recombinant fragments were acquired to investigate growth suppression; however, the C-terminus fragment did not indicate any growth suppression. This result is consistent with earlier literature that the C-terminus fragment cannot bind to the ROBO receptor [21,22]. The N-terminus SLIT3 not only induced growth suppression in four TNBC lines of MDA-MB-231, Hs578T, BT549, and HCC1937, but also increased ROBO2 expression, as shown in Figure 2. The ROBO2 receptor is downregulated in cancer cell lines compared to the non-malignant MCF10A, as shown in Supplementary Figure S4. Downregulation of ROBO2 in cancer cell lines is probably due to their survival mechanism. However, overexpression of ROBO2, as a result of incubation with SLIT3, suggests a positive feedback loop. A plausible mechanism, from neurobiology [23], could be that endocytosis of this ligand-receptor complex (a) enables the recycling of the receptor to the cell surface and (b) induces positive activation of ROBO signaling; hence, a positive feedback loop. Another plausible mechanism is from Slit2-mediated anti-tumoral function in colorectal cancer cells, where both SLIT2 and ROBO are downregulated [24]. In these cancer cells, (a) the Slit2 gene promoter is hypermethylated to suppress its expression, and (b) ROBO expression is downregulated through increased ubiquitin-mediated degradation. However, when the recombinant Slit2 protein is added to cancer cells, Slit2 activates the expression of USP33, a deubiquitinating enzyme, which prevents ROBO from degradation and stabilizes the protein.

### 2.4. CD36 Expression in Primary CAF Is Reversible and Concomitant with the Elevation of Adipogenic Markers

**Figure 3:**
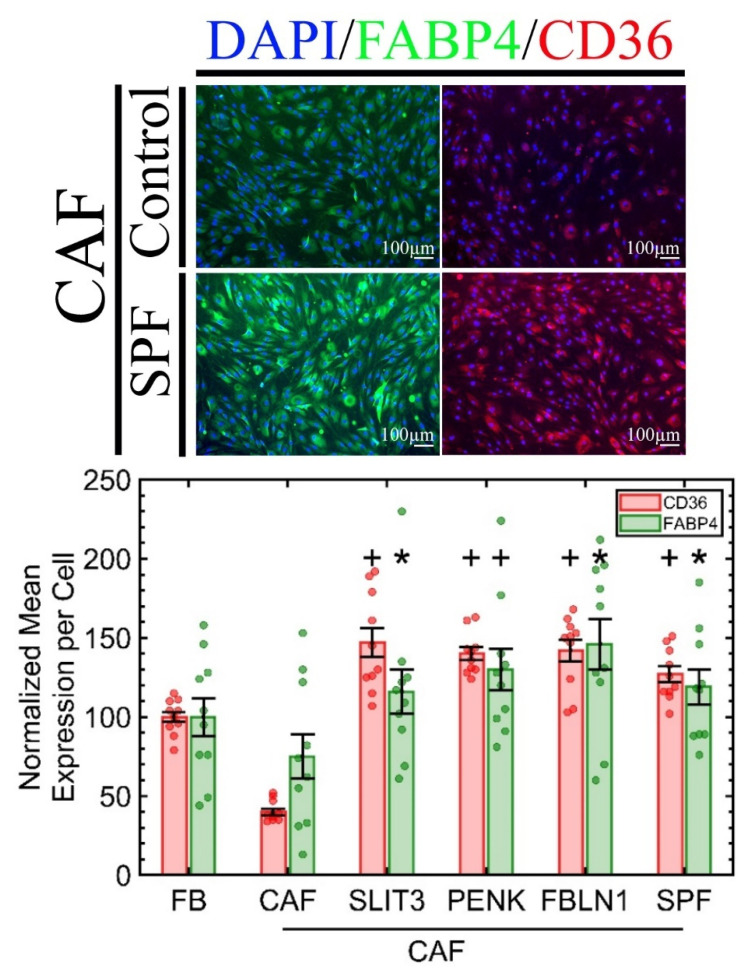
A CAF primary cell is reprogrammed by overexpressing CD36 and FABP4 when incubated with each of the recombinant proteins. The cocktail of RPs did not have a significant additive effect. Each condition had six replicates with at least two fields of view per well imaged. * *p*-value < 0.05 and + *p*-value < 0.001.

We have shown that CD36 is downregulated in primary FBs when cocultured with tumor epithelial cells or incubated with the recombinant protein activin A in a dose-dependent manner (2.25 ng/mL to 20 ng/mL) [10] with the proper controls (e.g., activin A neutralizing antibody). We also showed that (a) CD36^−^ FBs secrete more activin A, creating a positive feedback loop for tumor progression [10], and (b) CD36 expression in FBs is reversible once activin A is removed (Supplementary Results) [9]. However, within the tumor microenvironment, it is also important to show whether these three recombinant proteins, secreted from CD36^+^ FBs, can also revert CD36 in CAFs. We acquired CAFs (Cell Biologics: HC-6071 (Chicago, IL, USA)), which require the same culture medium as the primary FBs from Cell Biologics. To our knowledge, HC-6071 is the only commercially available mammary CAF. We incubated the CAFs with the same concentration of the RPs or their cocktail. The results, shown in Figure 3, indicate that CD36 expression is upregulated in CAFs as a result of exposure to each RP, but their cocktail (SPF) made no additional difference. Furthermore, CD36 is involved in fatty-acid transport and energy dissipation and is a marker for adipose tissue-derived stem cells [25,26]; hence, we hypothesized that one of the mechanisms of upregulation of CD36 in CAF, by RPs, should also be due to higher adipogenic markers, which is quantified by the overexpression of FABP4, per Figure 3. In adipocytes, FABP4, also known as aP2, is a carrier protein for fatty acids that is highly expressed in adipocytes and macrophages [27]. The controls were MCF10A and three TNBC breast cancer cell lines, including differentiated adipocytes that overexpress CD36 and FABP4, as shown in Supplementary Figure S5. The FABP4 is also expressed in MCF10A but not in TNBC cell lines, and the incubation of these lines with a cocktail of the three RPs did not alter the FABP4 expression. Hence, RPs only reverse FABP4 expression in the CAFs.

### 2.5. The Agonist Antibody for PENK Induces Growth Suppression in Four TNBC Lines

**Figure 4:**
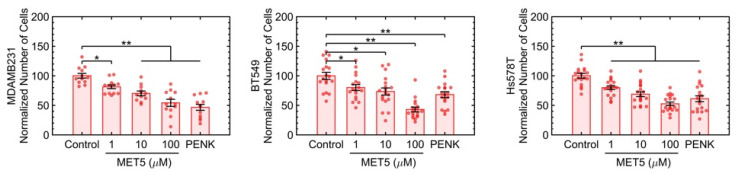
The agonist antibody for one of the PENK receptors, namely OGFr, induces growth suppression in all three TNBC cell lines. Each condition had six replicates with at least two fields of view per well imaged. * *p*-value<0.05 and ** *p*-value < 0.001.

Each of the three active ligands has at least one and up to five known receptors, and we opted to investigate [Met5] Enkephalin acetate salt hydrate, which is an agonist antibody for the OGFr, a receptor for PENK. The opioid growth factor (OGF) and its receptor, OGFr, regulate proliferation in normal and cancer cells, and their expression has been shown to be downregulated in ovarian cancer [28,29] and to restrict proliferation in pancreatic cancer [30]. OGF is chemically termed [Met(5)]-enkephalin, which is an endogenous opioid peptide that interfaces with OGFr and delays the cell cycle. The OGF-OGFr axis is also shown to induce growth suppression in both human breast [31] and pancreatic cells [32]. Figure 4 indicates that [Met(5)]-enkephalin induces growth suppression in three TNBC lines of MDA-MB-231, BT549, and Hs578t in a dose-dependent manner in 3D cultures. The control included colony formation in MCF10A, which indicated no loss of colony formation at the highest concentration of Met5, as shown in Supplementary Figures S6 and S7.

## 3. Discussion

In this manuscript, we reported an extended study on the growth suppression of multiple cancer cell lines with ligands derived from CD36^+^ fibroblasts or an agonist for one of the ligands, PENK. More importantly, we demonstrated that after treatment with these ligands, adipogenic makers in CAFs were elevated. Below, we discuss potential mechanistic insights for the tumor suppressive roles of these ligand/receptor pathways.

The SLIT/ROBO signaling has been extensively reviewed as a tumor suppressive pathway [33,34], where their expression is downmodulated in most cancer types, probably due to their survival mechanism. Nevertheless, their roles in cancer cell motility remain controversial. One study reports that this pathway inhibits cell migration and invasion by regulating E-cadherin-dependent adhesion and, consequently, β-catenin [35], while another study reports the opposite [34]. Such a contradiction is likely due to the complexities of multiple isoforms of SLIT (i.e., SLIT1-3) and ROBO (i.e., ROBO1-4) that might play different roles. Here, we have shown the N-terminal SLIT3 protein induces growth suppression of four triple-negative breast cancer cell lines. We have shown that ectopic SLIT3 treatment of cancer cells elevates the expression of the receptor ROBO2, suggesting a positive feedback loop. Plausible mechanisms include endocytosis of this ligand-receptor complex that enables the recycling of the complex to the cell surface to amplify ROBO signaling [23].

The roles of the glycoprotein FBLN-1 in tumors have not been extensively explored, and the results are somehow controversial. One study shows that overexpression of FBLN-1 in breast cancer cell lines promotes resistance to doxorubicin [36], whereas another study reports that inhibition of FBLN-1 in cancer cell lines increases the sensitivity to the same drug [37]. It is also reported that FBLN-1 interacts with the protumor ADAMTS-1 to block the activity of the latter in promoting the proliferation and migration of breast cancer cells [38]. Because of the scarcity of literature on the role of the ectopic FBLN-1 protein in the growth suppression of breast cancer cells, this subject matter may open up a new direction for cancer research.

PENK is proposed as a tumor suppressor in gastrointestinal stromal tumors. PENK expression negatively correlates with the tumor grade, and its high expression is linked to a favorable clinical outcome [39]. In another study, PENK is found to be downregulated in osteosarcoma (OS). Overexpression of PENK inhibits migration of OS cells, possibly through downregulation of the PI3K/Akt signaling pathway [40]. Here, we also have shown that the ectopic PENK protein or its agonist antibody also induces growth suppression of multiple triple-negative cell lines.

One of the major concerns in administering recombinant proteins or their agonist antibodies is their interactions with the immune system. A recent study indicates that CD36 expression positively correlates with the immune and stromal scores of different types of cancer [41]. Among factors secreted from the CD36^+^ FBs, (a) SLIT3 has been shown to increase the directional migration of monocytes and recruitment of myeloid cells in vivo [42]. (b) FBLN-1 is also found to be a pro-immunogenic glycoprotein involved in interactions between dendritic cells and cytotoxic T cells, and the high expression is linked to better lymphoid infiltration of breast tumors [43]. (c) PENK is involved in the activation of opioid receptors, which are highly expressed in the nervous system as well as immune cells [44]. The expression levels of PENK are also similar between the nervous and lymphoid systems, suggesting that this signaling pathway plays essential roles in both nervous and lymphoid systems [45]. In fact, it has been suggested that there is a reciprocal interaction between the immune system and opioids [46]. Collectively, there is clear evidence that these three ligands interact with the immune system, and their therapeutic use may potentially complement immunotherapy.

Our future efforts will focus on ex vivo tumor samples, where they will be incubated with the three recombinant proteins, sectioned, and stained for different cell types and quantifying their expression, e.g., vimentin, α-SMA, CD8, CD14. Next, the efficacy of the recombinant proteins will be determined in plasma, followed by in vivo experiments to assess tumor response in the mouse fat pad.

In conclusion, our current results strongly suggest that the proposed three ligands can induce growth suppression in breast cancer cells with minimal effect on healthy cells while reprogramming the tumor stroma. Moreover, these three ligands have the potential to interact with the immune system and complement immunotherapy.

## 4. Materials and Methods

### 4.1. Cell Culture

Epithelial cells (MDA-MB-231, BT-549, Hs578T, and HCC1937) were cultured in 96-well plates (3–6 repeats) using the 3D on-top method with respective growth media [47]. Briefly, a thin layer of Matrigel (17–20 µL/cm^2^, Corning 356243 (Corning, NY, USA)) was spread evenly on the surface of each well of a pre-chilled plate and incubated at 37 °C for 10 min to gel. Cells were suspended in the culture medium containing 5% Matrigel at a seeding density of 10,000 cells/cm^2^ for TNBC lines and 20,000 cells/cm^2^ for the non-malignant cell line unless otherwise specified. Cell suspensions were added to each well on the base Matrigel layer and incubated in a 37 °C humidified chamber for 24 h. The next day (considered as day 0), the culture medium was replaced by the treatment medium (described later in detail for each experiment) and thenceforth replenished with a fresh treatment medium after 48 h. On day 4 of the experiments, unless otherwise specified, the plates were washed with PBS three times and fixed with 4% fresh PFA, followed by DAPI staining and quantitative analysis.

Pre-adipocytes (ATCC PCS-210-010 (Manassas, VA, USA)) were cultured in fibroblast basal medium (ATCC PCS-201-030) supplemented with a low-serum fibroblast growth kit (PCS-201-041) as per ATCC’s handling information. The pre-adipocytes were cultured in adipogenic base media (R&D systems CCM007 (Minneapolis, MN, USA)) supplemented with adipogenic supplement (R&D system CCM011) and 10% FBS (ATCC 30-2020) for adipogenic differentiation.

Primary fibroblasts and cancer-associated fibroblasts (Cell Biologics H-6071 and HC-6071) were cultured in a complete fibroblast medium (Cell Biologics M2267) as per the company’s protocol.

### 4.2. Cell Culture Treatment

Human recombinant proteins, SLIT3 (Novus Biological 9255-SL-050 (Littleton, CO, USA)), FBLN1 (Abbexa abx066632), and PENK (Abbexa abx650333), were commercially acquired. (MET5) Enkephalin (Sigma M6638 ((St. Louis, MI, USA))) was also purchased commercially. For drug treatment on cells, each protein was used individually or combined (SPF). On day 0, the final concentrations of SLIT3, FBLN1, and PENK at 74 nM, 140 nM, and 33 nM, respectively, were added to the culture. MET5 was added at final concentrations of 1, 10, and 100 µM. Normal growth media were used as a control. All drugs and media were replaced every two days. The samples were fixed after four days of drug exposure.

To validate the growth inhibitory effect of protein ligands, neutralizing antibodies for SLIT3 (Novus Biological AF3629-SP), FBLN1 (Novus Biological NBP1-84725-25ul), and PENK (Novus Biological NBP1-90944-25ul), at a concentration of 1 µg/mL, were added to the CD36^+^ CM and applied to 3D-cultured cell lines.

### 4.3. Immunofluorescence Staining

For CD36 and FABP4 staining on fibroblasts, cell cultures were washed three times with DPBS (with Ca^2+^ and Mg^2+^, Thermo Scientific 14040-133 (Waltham, MA, USA)) and fixed at room temperature in 4% PFA (Thermo Scientific 28908) for 15 min. After three PBS washes, cells were permeabilized using a Triton X-100 solution (0.5%, Sigma T8787) for 10 min and then incubated for 1 h in a blocking solution containing 1% bovine serum albumin (BSA, Sigma A7638) in DPBS on a shaker at RT. The primary antibody, listed in Table 1, was diluted in the blocking solution and applied to cells overnight at 4 °C. The following day, samples were washed three times in DPBS (15 min per wash). Each secondary antibody, listed in Table 1, was diluted in the blocking solution and applied to samples for 1 h. Cells were washed three times in PBS (15 min per wash). Finally, the nuclei were counterstained with 100 ng/mL 4′-6-diamidino-2-phenylindole (DAPI, Life Science Technology D1306 (Seoul, Korea)).

For ROBO2 and FABP4 (Table 1) staining on epithelial cells, the same procedure was followed, except for replacing DPBS with PBS (without Ca^2+^ and Mg^2+^, Thermo Scientific 10010023).

### 4.4. Fluorescence Microscopy and Quantitative Analysis

The readout for each molecular endpoint is based on fluorescence microscopy, where our lab has excelled in the development of quantitative assays [48,49,50,51]. Typically, 60 to 300 cells are present per field of view with a 10× objective, which provides significant power for data analysis. On average, two to five fields of view are imaged per well, and there are three to six wells sampled per condition. Samples were imaged with an EVOS FL Auto Imaging System equipped with an AMEP 4633 10× phase objective (0.25 of NA and 6.9 mm of WD) and a 40× objective (0.8 ND and 3.3mm working distance). The excitation lasers were set at 385, 488, and 568 nm for DAPI, Alexa 488, and 568 fluorophores, respectively. All other imaging parameters were kept constant for all specimens.

### 4.5. Statistical Analysis

Most of the experiments were performed with six biological replicates, i.e., six wells per condition, where each well is imaged with a 10× objective and fluorescent microscopy. From each well, at least two fields of view are captured, cells are segmented using the DAPI stain and are counted, and relevant protein contents are computed on a cell-by-cell basis [48,49,50,51]. This information is then averaged over each field of view. The averaged results per field of view are then shown in the scatter bar chart, where each point in a bar chart represents either the total number of cells per field of view or the average fluorescent per cell per field of view. The error bar corresponds to the standard error of the mean for all fields of view and replicates per condition. Differences between groups were identified using Student’s *t*-test, and their significance is displayed with either one or two asterisks.

## Figures and Tables

**Table 1:** Details of the immunofluorescent staining for each molecular endpoint.

Target	CD36	ROBO2	FABP4
Permeabilization (Triton X-100)	0.5%	0.5%	0.5%
Blocking Solution (BSA)	1%	1%	1%
Primary Antibody	Novus Biological NB 400-144	Novus Biological NBP1-81399	R&D Systems AF3150
	1:250	1:250	1:250
Secondary Antibody	Abcam Ab175471	Abcam Ab150077	Novus Biological NB710-58353
	1:250	1:250	1:250

## Data Availability

Not applicable.

## Supplementary Materials

The following supporting information can be downloaded at: https://www.mdpi.com/article/10.3390/ijms232112744/s1.

## References

[1] Alexander J., Cukierman E. (2016). Stromal dynamic reciprocity in cancer: Intricacies of fibroblastic-ECM interactions. Curr. Opin. Cell Biol..

[2] Bissell M.J., Hall H.G., Parry G. (1982). How does the extracellular matrix direct gene expression?. J. Theor. Biol..

[3] Siemann D. (2011). Tumor Microenvironment.

[4] Kalluri R. (2016). The biology and function of fibroblasts in cancer. Nat. Rev. Cancer.

[5] Gascard P., Tlsty T.D. (2016). Carcinoma-associated fibroblasts: Orchestrating the composition of malignancy. Genes Dev..

[6] Peiris-Pages M., Smith D.L., Gyorffy B., Sotgia F., Lisanti M.P. (2015). Proteomic identification of prognostic tumour biomarkers, using chemotherapy-induced cancer-associated fibroblasts. Aging.

[7] Han C., Liu T., Yin R. (2020). Biomarkers for cancer-associated fibroblasts. Biomark. Res..

[8] Bukowski K., Kciuk M., Kontek R. (2020). Mechanisms of Multidrug Resistance in Cancer Chemotherapy. Int. J. Mol. Sci..

[9] Jabbari K., Winkelmaier G., Andersen C., Yaswen P., Quilici D., Furuta S., Cheng Q., Parvin B. (2021). Protein Ligands in the Secretome of CD36+ Fibroblasts Induce Growth Suppression in a Subset of Breast Cancer Cell Lines. Cancers.

[10] Cheng Q., Jabbari K., Winkelmaier G., Andersen C., Yaswen P., Khoshdeli M., Parvin B. (2020). Overexpression of CD36 in mammary fibroblasts suppresses colony growth in breast cancer cell lines. Biochem. Biophys. Res. Commun..

[11] Alkasalias T., Moyano-Galceran L., Arsenian-Henriksson M., Lehti K. (2018). Fibroblasts in the Tumor Microenvironment: Shield or Spear?. Int. J. Mol. Sci..

[12] Romer A.M., Luhr I., Klein A., Friedl A., Sebens S., Rosel F., Arnold N., Strauss A., Jonat W., Bauer M. (2013). Normal mammary fibroblasts induce reversion of the malignant phenotype in human primary breast cancer. Anticancer Res..

[13] DeFilippis R.A., Chang H., Dumont N., Rabban J.T., Chen Y.Y., Fontenay G.V., Berman H.K., Gauthier M.L., Zhao J., Hu D. (2012). CD36 repression activates a multicellular stromal program shared by high mammographic density and tumor tissues. Cancer Discov..

[14] Ligorio F., Di Cosimo S., Verderio P., Ciniselli C.M., Pizzamiglio S., Castagnoli L., Dugo M., Galbardi B., Salgado R., Loi S. (2022). Predictive role of CD36 expression in HER2-positive breast cancer patients receiving neoadjuvant trastuzumab. J. Natl. Cancer Inst..

[15] Neve R.M., Chin K., Fridlyand J., Yeh J., Baehner F.L., Fevr T., Clark L., Bayani N., Coppe J.P., Tong F. (2006). A collection of breast cancer cell lines for the study of functionally distinct cancer subtypes. Cancer Cell.

[16] Han J., Chang H., Giricz O., Lee G.Y., Baehner F.L., Gray J.W., Bissell M.J., Kenny P.A., Parvin B. (2010). Molecular predictors of 3D morphogenesis by breast cancer cell lines in 3D culture. PLoS Comput. Biol..

[17] Somasundaram V., Hemalatha S.K., Pal K., Sinha S., Nair A.S., Mukhopadhyay D., Srinivas P. (2016). Selective mode of action of plumbagin through BRCA1 deficient breast cancer stem cells. BMC Cancer.

[18] Katoh Y., Katoh M. (2005). Comparative genomics on SLIT1, SLIT2, and SLIT3 orthologs. Oncol. Rep..

[19] Condac E., Strachan H., Gutierrez-Sanchez G., Brainard B., Giese C., Heiss C., Johnson D., Azadi P., Bergmann C., Orlando R. (2012). The C-terminal fragment of axon guidance molecule Slit3 binds heparin and neutralizes heparin’s anticoagulant activity. Glycobiology.

[20] Kidd T., Brose K., Mitchell K.J., Fetter R.D., Tessier-Lavigne M., Goodman C.S., Tear G. (1998). Roundabout controls axon crossing of the CNS midline and defines a novel subfamily of evolutionarily conserved guidance receptors. Cell.

[21] Nguyen Ba-Charvet K.T., Brose K., Ma L., Wang K.H., Marillat V., Sotelo C., Tessier-Lavigne M., Chedotal A. (2001). Diversity and specificity of actions of Slit2 proteolytic fragments in axon guidance. J. Neurosci..

[22] Tong M., Jun T., Nie Y., Hao J., Fan D. (2019). The Role of the Slit/Robo Signaling Pathway. J. Cancer.

[23] Chance R.K., Bashaw G.J. (2015). Slit-Dependent Endocytic Trafficking of the Robo Receptor Is Required for Son of Sevenless Recruitment and Midline Axon Repulsion. PLoS Genet..

[24] Huang Z., Wen P., Kong R., Cheng H., Zhang B., Quan C., Bian Z., Chen M., Zhang Z., Chen X. (2015). USP33 mediates Slit-Robo signaling in inhibiting colorectal cancer cell migration. Int. J. Cancer.

[25] Achari A.E., Jain S.K. (2017). Adiponectin, a Therapeutic Target for Obesity, Diabetes, and Endothelial Dysfunction. Int. J. Mol. Sci..

[26] Gao H., Volat F., Sandhow L., Galitzky J., Nguyen T., Esteve D., Astrom G., Mejhert N., Ledoux S., Thalamas C. (2017). CD36 Is a Marker of Human Adipocyte Progenitors with Pronounced Adipogenic and Triglyceride Accumulation Potential. Stem Cells.

[27] Krusinova E., Pelikanova T. (2008). Fatty acid binding proteins in adipose tissue: A promising link between metabolic syndrome and atherosclerosis?. Diabetes Res. Clin. Pract..

[28] Fanning J., Hossler C.A., Kesterson J.P., Donahue R.N., McLaughlin P.J., Zagon I.S. (2012). Expression of the opioid growth factor-opioid growth factor receptor axis in human ovarian cancer. Gynecol. Oncol..

[29] Zagon I.S., Donahue R., McLaughlin P.J. (2013). Targeting the opioid growth factor: Opioid growth factor receptor axis for treatment of human ovarian cancer. Exp. Biol. Med..

[30] Cheng F., McLaughlin P.J., Verderame M.F., Zagon I.S. (2008). The OGF-OGFr axis utilizes the p21 pathway to restrict progression of human pancreatic cancer. Mol. Cancer.

[31] Zagon I.S., Porterfield N.K., McLaughlin P.J. (2013). Opioid growth factor—Opioid growth factor receptor axis inhibits proliferation of triple negative breast cancer. Exp. Biol. Med..

[32] Zagon I.S., Verderame M.F., Hankins J., McLaughlin P.J. (2007). Overexpression of the opioid growth factor receptor potentiates growth inhibition in human pancreatic cancer cells. Int. J. Oncol..

[33] Gara R.K., Kumari S., Ganju A., Yallapu M.M., Jaggi M., Chauhan S.C. (2015). Slit/Robo pathway: A promising therapeutic target for cancer. Drug Discov. Today.

[34] Jiang Z., Liang G., Xiao Y., Qin T., Chen X., Wu E., Ma Q., Wang Z. (2019). Targeting the SLIT/ROBO pathway in tumor progression: Molecular mechanisms and therapeutic perspectives. Ther. Adv. Med. Oncol..

[35] Prasad A., Fernandis A.Z., Rao Y., Ganju R.K. (2004). Slit protein-mediated inhibition of CXCR4-induced chemotactic and chemoinvasive signaling pathways in breast cancer cells. J. Biol. Chem..

[36] Pupa S.M., Giuffre S., Castiglioni F., Bertola L., Cantu M., Bongarzone I., Baldassari P., Mortarini R., Argraves W.S., Anichini A. (2007). Regulation of breast cancer response to chemotherapy by fibulin-1. Cancer Res..

[37] Lee N.V., Rodriguez-Manzaneque J.C., Thai S.N.M., Twal W.O., Luque A., Lyons K.M., Argraves W.S., Iruela-Arispe M.L. (2005). Fibulin-1 acts as a cofactor for the matrix metalloprotease ADAMTS-1. J. Biol. Chem..

[38] Mohamedi Y., Fontanil T., Cobo T., Vega J.A., Cobo J., Garcia-Suarez O., Cal S., Obaya A.J. (2019). The molecular interaction of ADAMTS-1 and fibulin-1 and its potential contribution to breast cancer biology. J. Cancer Metastasis Treat..

[39] Tang D.F., Lin T.L., Wang Y.Y., Cao H. (2019). High expression of proenkephalin is associated with favorable outcomes in patients with gastrointestinal stromal tumors. Cancer Manag. Res..

[40] Zhang H.P., Yu Z.L., Wu B.B., Sun F.R. (2020). PENK inhibits osteosarcoma cell migration by activating the PI3K/Akt signaling pathway. J. Orthop. Surg. Res..

[41] Chen Y.J., Liao W.X., Huang S.Z., Yu Y.F., Wen J.Y., Chen J., Lin D.G., Wu X.Y., Jiang N., Li X. (2021). Prognostic and immunological role of CD36: A pan-cancer analysis. J. Cancer.

[42] Geutskens S.B., Hordijk P.L., van Hennik P.B. (2010). The chemorepellent Slit3 promotes monocyte migration. J. Immunol..

[43] Pupa S.M., Argraves S.W., Forti S., Casalini P., Berno V., Agresti R., Aiello P., Invernizzi A., Baldassari P., Otwal W. (2004). Immunological and pathobiological roles of fibulin-1 in breast cancer. Oncogene.

[44] Machelska H., Celik M.O. (2020). Opioid Receptors in Immune and Glial Cells-Implications for Pain Control. Front. Immunol..

[45] Ovadia H., Magenheim Y., Behar O., Rosen H. (1996). Molecular characterization of immune derived proenkephalin mRNA and the involvement of the adrenergic system in its expression in rat lymphoid cells. J. Neuroimmunol..

[46] Plein L.M., Rittner H.L. (2018). Opioids and the immune system—Friend or foe. Br. J. Pharmacol..

[47] Lee G.Y., Kenny P.A., Lee E.H., Bissell M.J. (2007). Three-dimensional culture models of normal and malignant breast epithelial cells. Nat. Methods.

[48] Winkelmaier G., Parvin B. (2021). An enhanced loss function simplifies the deep learning model for characterizing the 3D organoid models. Bioinformatics.

[49] Cheng Q., Khoshdeli M., Ferguson B.S., Jabbari K., Zang C., Parvin B. (2020). YY1 is a Cis-regulator in the organoid models of high mammographic density. Bioinformatics.

[50] Khoshdeli M., Winkelmaier G., Parvin B. (2019). Deep Fusion of Contextual and Object-based Representations for Delineation of Multiple Nuclear Phenotypes. Bioinformatics.

[51] Chang H., Wen Q., Parvin B. (2015). Coupled Segmentation of Nuclear and Membrane-bound Macromolecules through Voting and Multiphase Level Set. Pattern Recognit..

