# Supplementary material for: CD36+ Fibroblasts Secrete Protein Ligands That Growth-Suppress Triple-Negative Breast Cancer Cells While Elevating Adipogenic Markers for a Model of Cancer-Associated Fibroblast
Source: Int J Mol Sci. 2022 Oct 22;23(21):12744. doi: 10.3390/ijms232112744 (PMC9654220; doi:10.3390/ijms232112744)
Supplement: Supplementary file 1 [file ijms-23-12744-s001.zip › ijms-1909226-supplementary.pdf]

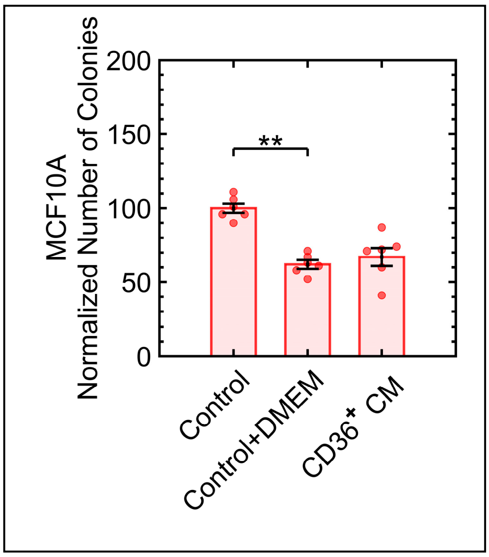

Supplementary Figure S1: Colony formation for MCF10A is marginally attenuated with the addition of the conditioned medium (CM) from the CD36$^+$ fibroblasts. However, this attenuation is partly due to the culture medium. Control: normal culture media for MCF10A (e.g, DMEM/F12 and supplements). Control+DMEM: Equal volume of DMEM is added to the CM of MCF10A. CD36$^+$ CM: CM of CD36$^+$ FB added to the standard CM of MCF10A, i.e., control. **: p-value<0.001

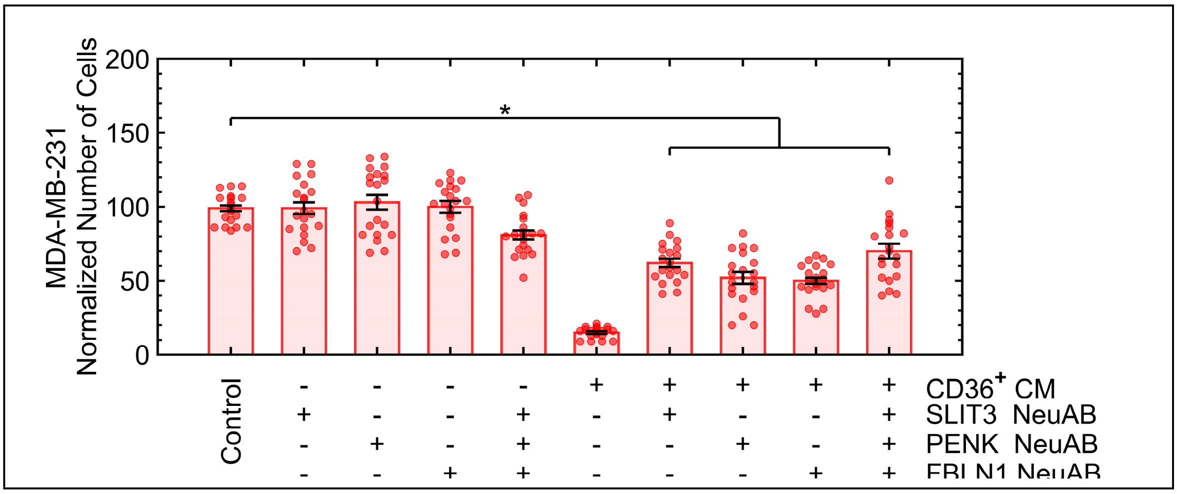

Supplementary Figure S2: Each neutralizing antibody attenuates growth suppression in MDA-MB-231 when added to the conditioned medium of CD36$^+$ fibroblasts. Hence, multiple factors in the secretome of CD36$^+$ FBs contribute to growth suppression. The number of cells per condition was automatically quantified from samples imaged through fluorescent microscopy. *: p-value <0.05

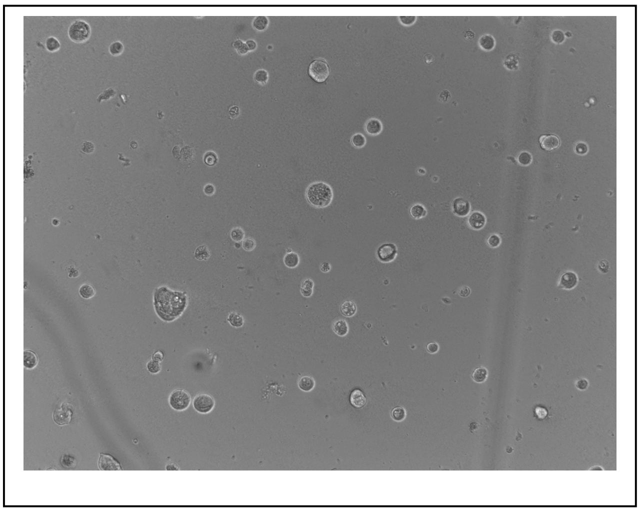

Supplementary Figure S3: HCC1937, with BRCA mutation, forms round colonies in 3D culture.

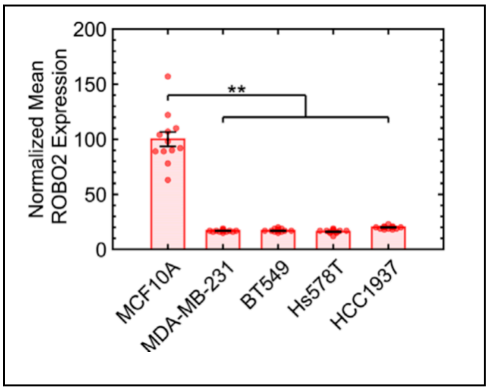

Supplementary Figure S4: ROBO2 receptor is down-regulated in cancer cell lines when compared with the non-transformed MCF10A cell line. **: p-value < 0.001

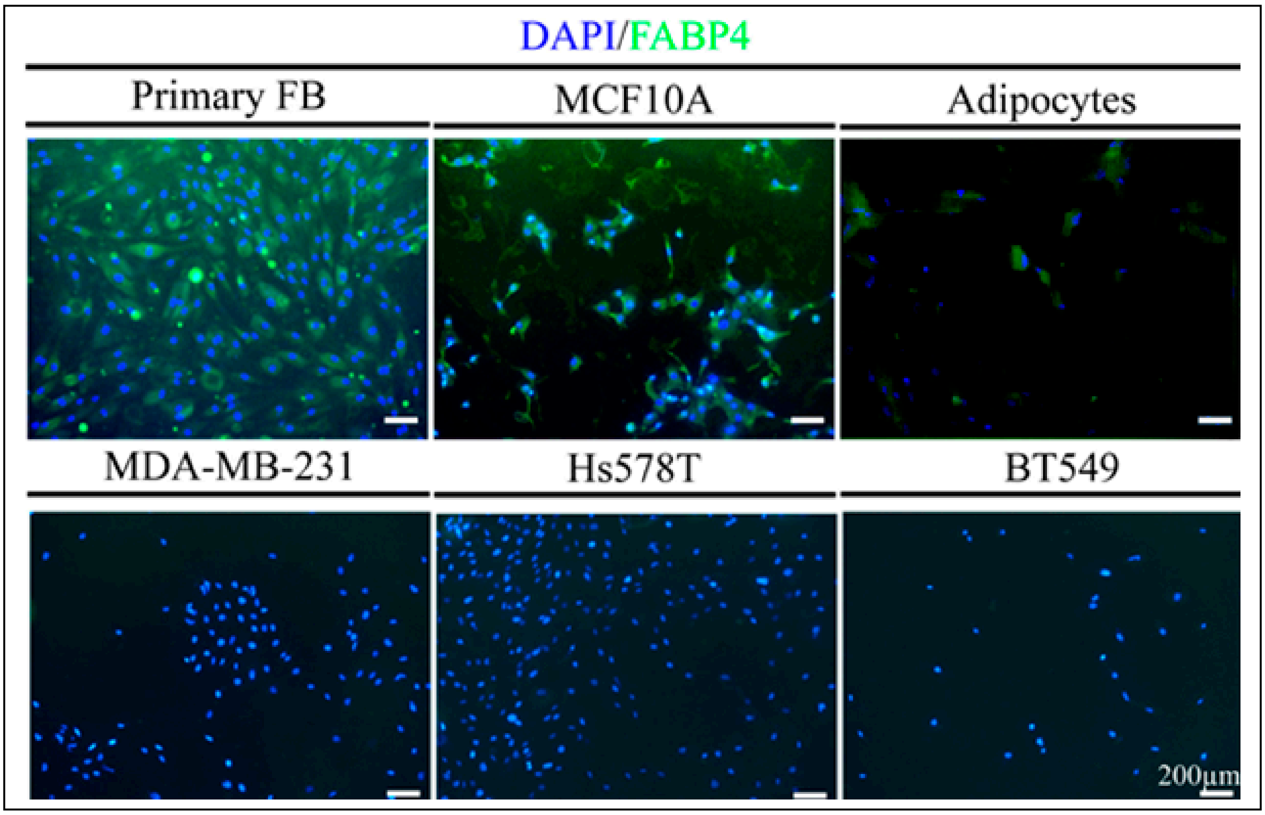

Supplementary Figure S5: FABP4 is expressed in primary FB and MCF10A but not in TNBC lines. The control is adipocyte cells.

Incubation of MCF10A or TNBC with a cocktail of the three recombinant proteins did not alter the FABP4 expression.

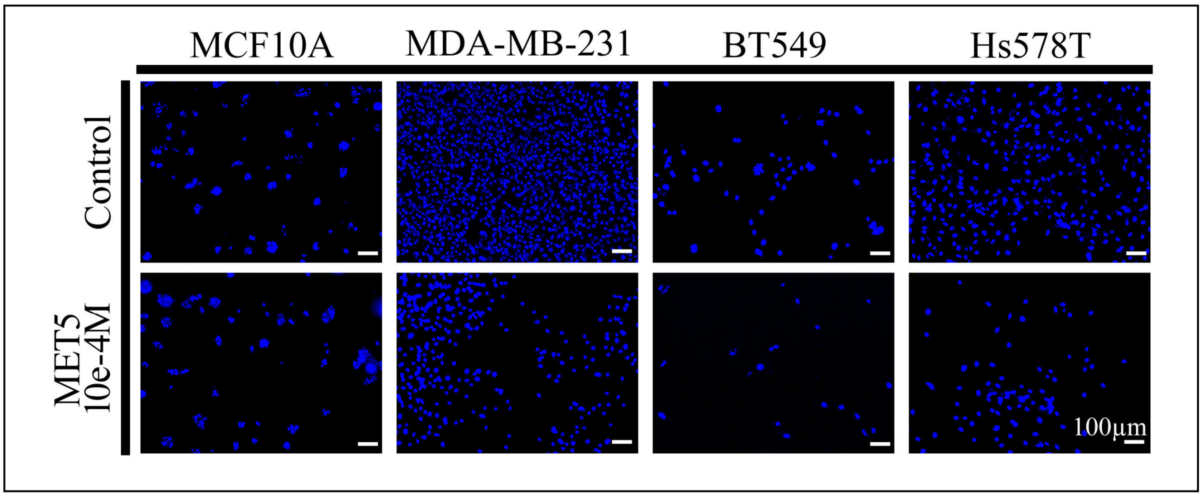

Supplementary Figure S6: Met5 induces growth suppression in TNBC lines but not in non-transformed MCF10A line

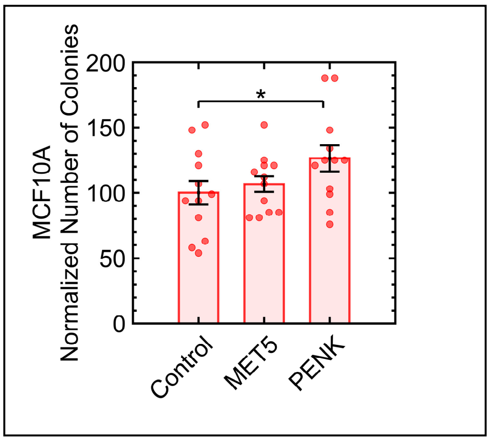

Supplementary Figure S7: Met5 and PENK do not induce colony suppression in MCF10A. However, there is a slight increase in the frequency of colony formation with PENK. *: p-value <0.05
